# Analysis of Medical Liability Lawsuits Involving Cardiologists in Greece

**DOI:** 10.7759/cureus.93305

**Published:** 2025-09-26

**Authors:** Nikolaos Baxevanos, Vasiliki Tzouma, Lambros Tzoumas, Konstantinos Tzoumas, Marianna Korre, Georgios Papadopoulos, Christos Katsouras

**Affiliations:** 1 9th Department of Orthopedics, Metropolitan General Hospital, Athens, GRC; 2 Department of Anesthesiology, University Hospital of Ioannina, Ioannina, GRC; 3 2nd Department of Cardiology, University Hospital of Ioannina, Ioannina, GRC

**Keywords:** cardiologist, greece, lawsuit, medical liability, negligence

## Abstract

The analysis of court decisions on medical malpractice can provide a detailed picture of patient mortality and morbidity and the causes that led to medical malpractice. The purpose of our research is to analyze, through published court decisions, cases of alleged medical errors by cardiologists in Greece. This is a retrospective study. Court decisions on medical malpractice by specialists and residents in cardiology from 1990 to 2024 were searched in Greek legal banks. The causes of manslaughter and bodily harm by negligence and the criteria for attributing liability were recorded. Forty-six court cases involving cardiologists were found, including 27 (58.7%) convictions and 19 (41.3%) acquittals - rejections of compensation. Among convictions, 25 (92.59%) involved manslaughter. These decisions concerned 27 patients (11 women and 16 men), with a mean age of 51.82 ± 22.72 years (range 25-84 years). The average compensation amounts imposed were 128.224 ± 71,980 €. In criminal cases, prison sentences ranged from six to 30 months with a three-year suspension. The duration of the legal dispute ranged from three to 20 years (average 5.93 ± 3.35 years). The main causes of manslaughter and bodily harm due to negligence were myocardial infarction (n = 9, 33.33%) and ruptured thoracic and abdominal aortic aneurysm (n = 5, 18.5%). Complications were mainly due to incorrect or delayed diagnosis (n = 17, 62.96%), delayed treatment (n = 14, 51.85%), incorrect treatment (n = 9, 33.33%), and incomplete ordering of diagnostic tests (n = 7, 25.9%). The findings reveal that while cardiology is generally considered a low-risk specialty, when malpractice cases do occur, they are associated with severe outcomes - most notably, a high rate of manslaughter convictions. The most common clinical scenarios leading to legal liability involved acute coronary syndrome and aortic aneurysm rupture, with diagnostic errors, delayed or incorrect treatment, and failure to order necessary tests being the most frequent causes of adverse outcomes. The prolonged duration of litigation and the emotional and financial burden it imposes on both physicians and patients also underline the need for reforms that promote faster resolution and reinforce quality and safety in healthcare delivery.

## Introduction

Cardiology is classified as a low-risk specialty for medical malpractice liability and remains relatively protected from claims [1,2]. In another study of medical malpractice claims from 1986 to 2011, the percentage of claims involving cardiology was 1.4% [3] and was much lower than specialties such as traumatology and orthopedic surgery (15.7%) or obstetrics and gynecology (12.5%) [1].

In contrast, in the study by Mangalmurti et al., the annual rate of cardiologist malpractice lawsuits is higher (8.6%) than for physicians overall (7.4%). Among 530 claims for compensation, 72 (13.6%) resulted in a payment with an average compensation of $164,988, while the average defense cost for claims ranged from $72,901 to $83,593 [4]. In the study by Patel et al. (2010-2015), 166 cases were found, with more than half of the cases involving a cardiologist defendant. The most common reasons for litigation were treatment failure, failure to diagnose, failure to refer/order diagnostic tests, and patient death. The most frequently missed diagnosis was myocardial infarction [5]. In a study by Wu et al. from Japan, among 36 closed myocardial infarction malpractice claims, 20% of the cases were decided in favor of the plaintiffs. The average award was $100,639 ± $49,617. Cardiologists and emergency physicians were involved in 56.3% of the cases, but won 92.6% of the cases, while other specialists lost almost 25% of the cases [6].

Coronary artery disease is one of the leading causes of death worldwide [7]. Despite decades of medical advances, coronary angiography and percutaneous coronary intervention have remained the mainstay treatments for coronary artery disease. As the complexity of the procedure now includes broader indications of chronic total occlusion, calcified coronary artery disease, and cardiogenic shock [8], the likelihood of procedure-related complications may increase despite the procedures contributing to a better prognosis [7,9]. In the study by Oetgen et al., claims involving cardiac catheterization and coronary angioplasty accounted for 12% and 7% of closed cardiovascular claims, respectively. The average compensation payment was $248,291. The most common claim among closed cardiovascular claims was diagnostic error, and the most prevalent diagnosis was coronary atherosclerosis [7]. In another study of cardiac catheterization litigation, among 1,361 closed claims, 301 (22%) resulted in payments to the claimant with an average award of $230,987. The most common alleged error was improper performance (35.4%). Failure to perform an indicated procedure had the highest ratio of paid to closed claims (41%) with an average award of $246,988. In terms of injury severity, death was the most common outcome (44%) [10].

The analysis of court decisions on medical malpractice identifies the causes of complications and the criteria for convicting doctors, provides a detailed picture of patient mortality and morbidity, and has contributed significantly to the improvement of medical practice and patient safety. In Greece, there are no relevant publications for cardiologists. The purpose of this study is to systematically analyze, through published court decisions, cases of alleged medical errors by cardiologists in Greece between 1990 and 2024. Specifically, we aimed to (1) assess the frequency and outcomes of malpractice claims involving cardiologists, (2) identify the main clinical and organizational factors associated with liability, and (3) examine the medico-legal implications for both civil and criminal cases. Our findings are intended to inform cardiologists, hospital administrators, legal experts, and policy makers about recurrent patterns in malpractice litigation and to suggest potential reforms for clinical practice, medical education, and legal procedures.

## Materials and methods

The research sought court decisions of all levels and jurisdictions regarding the medical liability of specialist and subspecialist cardiologists at the criminal, civil, administrative, and disciplinary levels. The search included published court decisions of criminal, civil, administrative, and disciplinary content from 1990 to 2024 from the legal information banks “NOMOS” [11], “Sakkoulas online.gr” [12], and “Athens Bar Association Bank” [13], as well as from legal journals such as Nomiko Vima [14], Criminal Justice [15], Criminal Chronicles [16], and Armenopoulos Publications [17]. The keywords “cardiologist”, “medical liability”, “negligence”, and “medical error” were used.

Only finalized decisions (non-appealable) were included, and duplicate entries across databases were removed. Cases involving cardiologists as either primary defendants or co-defendants were included. Unpublished and pending cases were excluded. Two independent reviewers (a legal researcher and a medical expert) extracted data. A senior Emeritus Professor of Medicine reviewed all cases to ensure consistency in clinical interpretation. Disagreements were resolved by consensus. Extracted data included patient demographics, clinical setting (emergency vs. elective), type of legal proceeding (civil vs. criminal), number and specialty of defendants, type of outcome (conviction vs. acquittal; compensation vs. dismissal), compensation amounts, duration of litigation, and legal reasoning provided by the courts. The causes that led to the unfavorable outcome, homicides, bodily harm, and the criteria of the legal system for the conviction of the cardiologists were also recorded. It was also checked whether detailed history, informed consent of the patients, timely medical treatment, and continuous and mandatory monitoring were applied to all patients. It was also recorded on which alleged error each decision judges and when and how it attributes responsibility. The percentages of decisions, of cases in general, but also based on their distinctions as criminal convictions, civil awards of compensation, criminal acquittals, civil dismissals of compensation claims, the average values, the standard deviation, the median, and the range of compensation for cardiologists, specialists, and residents, were calculated. Clinical conditions were classified based on the primary cause of harm as stated in the court ruling, supplemented by medical expert interpretation. Where multiple conditions existed, the dominant cause of death or injury was prioritized. Legal criteria for liability (e.g., delayed diagnosis, incorrect treatment, and documentation failures) were taken directly from the text of judgments. Rare or single-occurrence criteria were grouped into broader categories to enhance interpretability. Residents and specialists were distinguished based on explicit court references. No ethical approval was required as only publicly available, finalized legal decisions were analyzed.

## Results

A total of 102 court decisions corresponding to 46 unique malpractice cases were identified. Among these, 27 (58.69%) resulted in convictions (criminal rulings or civil compensation awards) and 19 (41.3%) resulted in acquittals or dismissal of compensation claims. Of the 27 conviction cases, 25 (92.6%) involved manslaughter by negligence and two (7.4%) involved bodily harm by negligence. The criminal cases were 11 (40.74%), and the prison sentence imposed ranged from six to 30 months with a three-year suspension. The civil cases were 16 (59.26%) (Table 1). The convictions involved 11 women and 16 men. The mean age of patients was 51.82 ± 22.72 years with a range of 25 to 84 years.

**Table 1:** Characteristics of medical liability lawsuits involving cardiologists: convictions, criminal cases, civil cases, and acquittals - exonerations of compensation.

	Ν	%
Total cases	46	100%
Conviction cases	27	92.6%
Acquittals	19	41.3%
Manslaughter by negligence	25	92.6%
Bodily injuries by negligence	2	7.4%
Negligence by a cardiologist specialist	23	85.2%
Negligence by a resident	3	14.8%
Criminal cases	11	40.7%
Civil cases	16	59.3%

The duration of the legal dispute ranged from three to 20 years, and the compensation paid ranged from 57,000 to 300,000 € (Table 2).

**Table 2:** Average, standard deviation, and range of the duration of litigation in criminal and civil cases and compensation payments. SD: standard deviation

	Average ± SD	Range
Duration of litigation (years)	7.04 ± 4.3	3-20
Duration of criminal cases (years)	6.1 ± 3.38	3-11
Duration of civil cases (years)	8.46 ± 4.57	4-20
Compensation (Euro)	128,224 ± 71,980	57,000-300,000

Compensation was awarded against physicians directly, and in practice, these amounts are usually covered by malpractice insurance where applicable, though court rulings rarely specify the source. In total, 44 physicians were convicted across these cases, as multiple doctors were often co-defendants in a single case. Among them, 27 doctors were cardiologists, 11 were specialists in other specialties, and six were internists. Specialists accounted for 85.2% (n = 23) of negligent acts and residents for 14.8% (n = 4). Of the 25 manslaughter cases, 22 involved specialists and three involved residents. Most lawsuits were filed against individual physicians, though hospitals were occasionally named as co-defendants. The percentage of convicted resident doctors, either in independent operation or in collaboration with a specialist or second resident doctor, compared to the total number of convicted specialist doctors was 6.81%. Regarding the cases that resulted in criminal conviction or civil compensation, the percentage concerning resident doctors is 11.11%.

Table 3 shows the cases in which there was convergent liability, the causes, and the doctors involved.

**Table 3:** Cases in convergent liability and diseases.

Convergent liability of specialized doctors	Cases	Disease
Cardiologist	3	Cardiomyopathy, acute coronary syndrome, aortic arch separation
Cardiologist, internal medicine physician	1	Acute coronary syndrome
Cardiologist, nuclear medicine doctor	1	Acute coronary syndrome
Cardiologist, cardiac surgeon	1	Pneumonia
Cardiologist, general surgeon	1	Thoracic aortic rupture from a traffic accident
Cardiologist, internal medicine physician, 2 radiologists	1	Foreign body in the larynx
Cardiologist, internal medicine physician, rheumatologist	1	Endocarditis from a pacemaker lead
Convergent liability of specialized doctors and residents	Cases	Diseases
Resident of cardiology, general surgeon	1	Abdominal aortic aneurysm rupture
Resident of cardiology, internal medicine physician	1	Acute coronary syndrome
Convergent liability of residents	Cases	Diseases
Resident of cardiology, resident of general medicine	1	Airway obstruction in drunkenness

Informed consent was rarely mentioned in court decisions. In the few cases where it was documented, courts emphasized that informed consent does not absolve physicians of liability for negligent acts, particularly errors in diagnosis and treatment. In the court decisions analyzed in the research, there was no conviction of a doctor for the death or bodily harm of a patient when the doctor provided full information to the patient before the medical procedure and received the patient's corresponding written consent. The incomplete information of the patient in two cases was an aggravating factor in the conviction of the doctors, where negligence for the medical procedure was also imputed.

Most diagnostic errors occurred in the emergency department setting, with a notable proportion of cases (40.7%) involving patient presentation during evening or night hours. Of the 16 civil cases analyzed in the study, seven (43.75%) involved lawsuits against private clinics and cardiologists employed in them, and the remaining nine (56.25%) involved public hospitals. At the level of defendant private clinics and private doctors, the payment of the awarded compensation was made by the insurance companies, while public hospitals paid from their budgets.

A primary cardiovascular disease that caused a claim and conviction was identified in 20 (74.07%) cases. The most common condition was acute coronary syndrome (33.33%), followed by ruptured thoracic/abdominal aortic aneurysm (Table 4).

**Table 4:** Causes of manslaughter and bodily harm due to negligence of specialist and resident cardiologists.

Disease	Ν	%
Manslaughter by negligence		
Myocardial infarction	9	33.33%
Aneurysm rupture: thoracic-abdominal aorta	5	18.5%
Cardiomyopathy of pregnancy	1	3.7%
Sudden death of a football player	1	3.7%
Aortic valve stenosis	2	7.4%
Hypertrophic cardiomyopathy	1	3.7%
Iliac artery erosion in coronary angiography	1	3.7%
Pneumonia sepsis	1	3.7%
Airway obstruction in intoxication	1	3.7%
Foreign body in the larynx	1	3.7%
Pheochromocytoma	1	3.7%
Exercise test in myocardial ischemia	1	3.7%
Bodily injuries due to negligence		
Thoracic aortic rupture from a traffic accident	1	3.7%
Endocarditis	1	3.7%

In a case of manslaughter by negligence, the prosecution for joint liability of a cardiologist, obstetrician, and anesthesiologist was discontinued after 20 years of litigation. This was a case of a bicuspid aortic valve with severe stenosis in a pregnant woman, significant pulmonary hypertension, and reduced contractility. These findings did not concern the cardiologist, and the cesarean section was performed at the end of the pregnancy, when the patient's condition had deteriorated, under epidural anesthesia. In 11 (40.74%) cases, the patients were treated during the evening and night hours.

The main criteria of the legal order, alone or in combination, for the conviction of cardiologists, were incorrect or delayed diagnosis (62.96%), delayed treatment (51.85%), incorrect treatment (33.33%), and incomplete laboratory testing (25.9%) (Table 5).

**Table 5:** Legal criteria alone or in combination for the conviction of doctors.

Legal criteria	Ν	%
Wrong or late diagnosis	17	62.96%
Delayed treatment	14	51.85%
Incorrect treatment	9	33.33%
Insufficient laboratory testing	7	25.9%
Non-referral to a specialist	1	3.7%
Non-referral from a resident to a specialist	2	7.4%
Insufficient follow-up	2	7.4%
The specialist was content with the resident's diagnosis	1	3.7%
Non-performance of a history and physical examination by a specialist	2	7.4%
Refusal to perform coronary angiography	1	3.7%
Discharge	2	7.4%
Insufficient information	2	7.4%
Absence of a specialist or resident	1	7.4%

## Discussion

In the present study, we analyzed the characteristics of all criminal and civil cases of medical malpractice by cardiologists in Greece between 1990 and 2024. The analysis of published court decisions provided us with sufficient information regarding the clinical details of the allegations of medical malpractice by cardiologists.

Our analysis revealed a disproportionately high rate of manslaughter convictions among cardiologists in Greece compared to international literature, where malpractice claims more often involve compensation without criminal liability. This may be explained by cultural and systemic barriers to filing lawsuits for non-fatal complications, combined with the prolonged duration and high costs of litigation. Consequently, the cases reaching final judgment may disproportionately represent fatal outcomes.

Our research found 27 (58.6%) cases of medical malpractice by a specialist in 22 cases (81.48%) and a cardiologist or a specialist in five cases (18.52%). In 19 (41.4%) acquittal-exoneration cases, there was no connection between the treatment of the patient and the allegation of medical malpractice. The rate of manslaughter was 25 (92.6%), and the rate of bodily harm was 2 (7.4%). This high mortality from negligence that we described is likely to be fictitious and may be due to the lack of complaints and claims for mild or moderate complications, which would have increased the number of cases and reduced the mortality, as in other publications [6]. A possible explanation for this finding may be the difficulty of proving medical liability, the extremely long duration of a legal dispute in our country, and the cost of legal fees paid by patients. The doctor-patient relationship and the family may be responsible, but also the fact that cardiac patients are more psychologically prepared for adverse clinical outcomes that may arise, since it is widely known in society that the risk of heart disease is high, and therefore, they are less likely to file a lawsuit in these cases. The treatment of patients by specialist doctors contributed to the mortality, while physician burnout may also have played an important role, as 40.74% of patients were treated during the afternoon and night hours in the emergency department. Restrictions on physician working hours and understanding the role of organizational factors that contribute to and prevent medical errors may improve the quality of patient care [10].

In the study by Mangalmurti et al., mortality was 57.4%. Among 530 claims, only 13.6% resulted in a payment of compensation with a mean value of $164,988, while the mean defense cost for claims that resulted in payment was $83,593 ± $72,901 [4]. In another study, the out-of-court settlement rate was 30%. In that study, the total number of claims and annual compensation payments in cardiology had increased overall from 2006 to 2015 [18].

The main diseases responsible for 55.55% of serious injuries were acute coronary syndrome (33.33%) and ruptured thoracic/abdominal aortic aneurysm (22.22%). The observed mortality and physical injuries were due to incorrect or delayed diagnosis (62.96%), delayed treatment (51.85%), and incorrect treatment (33.33%). Diagnostic errors are the first and leading cause of claims and litigation and increase mortality and length of hospital stay in patients presenting to the emergency department [19,20]. The primary conditions leading to litigation - acute coronary syndrome and ruptured aortic aneurysms - mirror international patterns, but the rate of manslaughter rulings and extended litigation timelines appear unique to the Greek medico-legal context. These findings underscore the importance of timely and accurate diagnosis, particularly in emergency settings where physician fatigue and reduced staffing may contribute to errors.

Knowledge of the aspects of the incorrect diagnostic process can help and provide information for targeting interventions. In addition to the factors described for mortality, lack of experience, overconfidence of physicians, and failure to order appropriate diagnostic testing may have contributed to diagnostic error. In the study by Kachalia et al., the main causes of failure in the diagnostic process in the emergency department were failure to order an appropriate diagnostic test (58% of errors), failure to perform an adequate medical history or physical examination (42%), misinterpretation of a diagnostic test (37%), and failure to order an appropriate consultation (33%). Incomplete follow-up, failure to perform a physical examination by a specialist, the specialist being content with the diagnosis of the resident, and disciplinary offenses, such as a cardiologist’s refusal to perform a coronary angiography and the specialist’s absence from work, also contributed to the diagnostic error in a total of 22.22% of the cases [21].

In our study, in 25.9% of the conviction cases, there was a failure to order or a delay in laboratory testing. In the Calder et al. study, with 368 (0.6%) cases of medical negligence by a cardiologist for the years 2009 to 2018, failure to order or a delay in ordering a diagnostic test was associated with a 4.1% mortality (N = 15). In 12 (80.0%) out of the 15 cases, the patient suffered serious harm or death [19]. In the study by Patel et al., failure to diagnose (69.3%), with myocardial infarction being the most common misdiagnosis, and failure to refer/order diagnostic tests was in 64.5% of cases, and patient death was 71.1% [5]. Failure to order an appropriate diagnostic test was the main cause of misdiagnosis in the second group of diseases, ruptured thoracic and abdominal aortic aneurysms. In the study by Mangalmurti et al., the incidence of failure to diagnose aortic dissection was 2.5% [4].

In our investigation, three cardiology residents and one general medicine resident were convicted at the criminal level. These were doctors at an advanced stage of their specialization who in two cases (acute myocardial infarction and aneurysm rupture) did not call the corresponding specialists, and in one case, there was inadequate monitoring of airway obstruction. In the legal framework of our country and in the operation of the residents in the public hospitals of the National Health System, in addition to the training provided, the residents provide medical services and perform medical procedures assigned to them, without the constant supervision/monitoring of a specialist, but they are obliged, depending on the severity of each incident, to call specialists. This highlights the need for structured supervision protocols and clearer delineation of responsibilities between residents and supervising physicians.

In our research, the lack of information provided to the patient significantly contributed to the criminal conviction of doctors in two cases. Written consent of the patient or his relatives, when he is not able to give consent, is of great importance when the information provided, which is written on the corresponding form by the doctor, is detailed and completely informative in understandable language for all possible complications of the medical act. Such written consent constitutes an important piece of evidence for the doctor's defense in his legal action with a relevant allegation. Courts consistently judged that while consent forms cover procedural risks, they do not excuse negligent acts, thus underscoring the importance of not only obtaining consent but also ensuring high diagnostic and treatment standards.

Hospital-level quality assurance systems could benefit from incorporating medico-legal data into routine morbidity and mortality reviews, focusing particularly on acute coronary syndrome and aortic emergencies. At a policy level, reforms are needed to shorten litigation times and improve diagnostic support tools in emergency cardiology.

Our study has several limitations. Although we used all existing legal information banks in Greece, these may not be representative at a national level. There is a significant number of unpublished cases, which is due, among other things, to the extremely long duration of litigation. Also, the sample size of the claims was limited. Only finalized, published court decisions were included, which may bias the sample toward severe cases with fatal outcomes. Many unpublished or pending cases, particularly those involving minor complications, were excluded. The retrospective design and reliance on legal summaries limit the depth of available clinical details. Additionally, court judgments may underreport the presence of informed consent and other documentation practices. However, despite the limitations of our research, our study was able to identify causes and criteria that led to complications and complaints during the performance of medical procedures by cardiologists. It is what cardiologists require to understand the details of previous lawsuits against fellow cardiologists, including the clinical contexts in which they are most likely to arise.

## Conclusions

This study provides the first systematic analysis of published court decisions involving cardiologists in Greece accused of medical malpractice over a 34-year period. The findings reveal that while cardiology is generally considered a low-risk specialty, when malpractice cases do occur, they are associated with severe outcomes - most notably, a high rate of manslaughter convictions. The most common clinical scenarios leading to legal liability involved acute coronary syndrome and aortic aneurysm rupture, with diagnostic errors, delayed or incorrect treatment, and failure to order necessary tests being the most frequent causes of adverse outcomes. The study highlights emphasize the importance of timely and accurate diagnosis, the necessity for proper supervision of residents, thorough documentation, and clear communication with patients. The prolonged duration of litigation and the emotional and financial burden it imposes on both physicians and patients also underline the need for reforms that promote faster resolution and reinforce quality and safety in healthcare delivery. Ultimately, understanding the clinical and legal dimensions of past malpractice cases can serve as an educational tool for cardiologists and healthcare institutions. It can guide risk management practices, inform policy development, and enhance the standard of care, ultimately leading to improved patient safety and reduced medico-legal exposure.
